# What Happens at Surfaces and Grain Boundaries of Halide Perovskites: Insights from Reactive Molecular Dynamics Simulations of CsPbI_3_

**DOI:** 10.1021/acsami.2c09239

**Published:** 2022-08-30

**Authors:** Mike Pols, Tobias Hilpert, Ivo A.W. Filot, Adri C.T. van Duin, Sofía Calero, Shuxia Tao

**Affiliations:** ^†^Materials Simulation & Modelling, Department of Applied Physics, ^‡^Center for Computational Energy Research, Department of Applied Physics, and ^§^Laboratory of Inorganic Materials Chemistry, Schuit Institute of Catalysis, Department of Chemical Engineering and Chemistry, Eindhoven University of Technology, 5600 MB, Eindhoven, The Netherlands; ⊥ Department of Mechanical Engineering, Pennsylvania State University, University Park, Pennsylvania 16802, United States

**Keywords:** ReaxFF, molecular dynamics, metal halide perovskite, degradation, stability, surfaces, grain boundary, defects

## Abstract

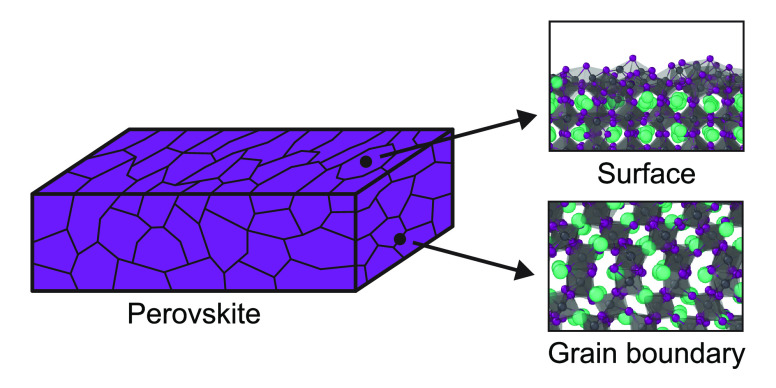

The commercialization of perovskite solar cells is hindered by the poor long-term stability of the metal halide perovskite (MHP) light-absorbing layer. Solution processing, the common fabrication method for MHPs, produces polycrystalline films with a wide variety of defects, such as point defects, surfaces, and grain boundaries. Although the optoelectronic effects of such defects have been widely studied, the evaluation of their impact on the long-term stability remains challenging. In particular, an understanding of the dynamics of degradation reactions at the atomistic scale is lacking. In this work, using reactive force field (ReaxFF) molecular dynamics simulations, we investigate the effects of defects, in the forms of surfaces, surface defects, and grain boundaries, on the stability of the inorganic halide perovskite CsPbI_3_. Our simulations establish a stability trend for a variety of surfaces, which correlates well with the occurrence of these surfaces in experiments. We find that a perovskite surface degrades by progressively changing the local geometry of PbI_*x*_ octahedra from corner- to edge- to face-sharing. Importantly, we find that Pb dangling bonds and the lack of steric hindrance of I species are two crucial factors that induce degradation reactions. Finally, we show that the stability of these surfaces can be modulated by adjusting their atomistic details, by either creating additional point defects or merging them to form grain boundaries. While in general additional defects, particularly when clustered, have a negative impact on the material stability, some grain boundaries have a stabilizing effect, primarily because of the additional steric hindrance.

## 1 Introduction

Metal halide perovskites (MHPs) with the ABX_3_ formula (A = organic or inorganic cation; B = metal cation; X = halide anion) have attracted a great deal of attention as low-cost and high-performance semiconductors for applications in photovoltaics^1,2^ and light-emitting diodes.^3,4^ MHPs are typically synthesized using facile solution-processing deposition techniques. Halide salts are mixed in solution with metal halide precursors, where solvent evaporation causes the formation of colloidal particles in solution, with further evaporation resulting in the growth of these particles in a polycrystalline perovskite film.^5−8^ This simple fabrication procedure offers a wide tunability in compositions and dimensions. However, it generally introduces a wide variety of defects in the material, ranging from point defects to crystal surfaces and grain boundaries.^9,10^ For the majority of these defects, it is found that they have a limited electronic effect on the MHP, with point defects,^11−13^ surfaces,^14,15^ and grain boundaries^16−19^ mainly resulting in electronically benign defect levels. While it is common knowledge that defects induce instability problems in MHPs, for instance through a defect-driven accumulation of defects at grain boundaries^20−22^ or material degradation at perovskite interfaces,^23^ an understanding of the dynamics of the degradation reactions at an atomistic scale and their impact on the long-term stability of the materials and devices is limited.

Until now, the common understanding in the literature has been that during the degradation of MHPs the material disintegrates into its precursors. Specifically, various experiments have demonstrated the formation of PbX_2_ and amorphous PbX_2–*x*_ (with X = I or Br) using X-ray diffraction (XRD) measurements in the degradation of MHPs.^24−28^ Moreover, metallic lead has also been found as a degradation product in MHPs.^27,28^ Using electron microscopy, it has been established that the degradation of halide perovskites tends to occur at surfaces and grain boundaries.^27,29,30^ For that reason, a variety of efforts have been devoted to minimizing the occurrence of such grain boundaries in perovskite films to enhance the stability of the materials and devices.^31,32^ However, despite this knowledge on the perovskite films and the decomposition products, a detailed understanding of the degradation pathways of halide perovskites is still lacking.

In this work, our objective is to provide insights into the role of surfaces and grain boundaries in the long-term stability of MHPs. To do so, we perform reactive molecular dynamics simulations using a reactive force field (ReaxFF) we developed for CsPbI_3_.^33^ This ReaxFF force field makes use of a dynamical bond order to describe the breaking and creation of bonds.^34−36^ The simulations allow us to characterize the evolution of the atomic species under thermal stress and to establish a stability trend of a variety of surfaces. Furthermore, based on the simulation trajectories, we establish what structural features make a surface stable or unstable and, in the case of decomposition, through which atomistic processes it proceeds. Finally, we find that additional point defects are generally detrimental to perovskite stability, whereas grain boundaries can have either positive or negative effects on the stability of the perovskite lattice.

## 2 Results and Discussion

In the following sections, we present our findings on the stability of the surfaces of inorganic CsPbI_3_. In section 2.1, we show the structural models of the surfaces and grain boundaries that we investigate in this work. We specifically highlight the equivalence between orthorhombic and cubic surface models. In section 2.2, before we show the defect-induced chemical instability, we first illustrate the effects of surfaces on the phase stability of CsPbI_3_ by comparing the structural details of the surfaces with those in the bulk. Section 2.3 evaluates the stability of different perovskite surfaces under thermal stress and elaborates on the mechanism of defect-induced degradation. We then explore the effects of additional defects located at surfaces by analyzing the stability of the surfaces of perovskites with defects in section 2.4. Finally, in section 2.5, we investigate the effects of grain boundaries on the stability of the perovskite lattice.

### 2.1 Structural Models

**Figure 1:**
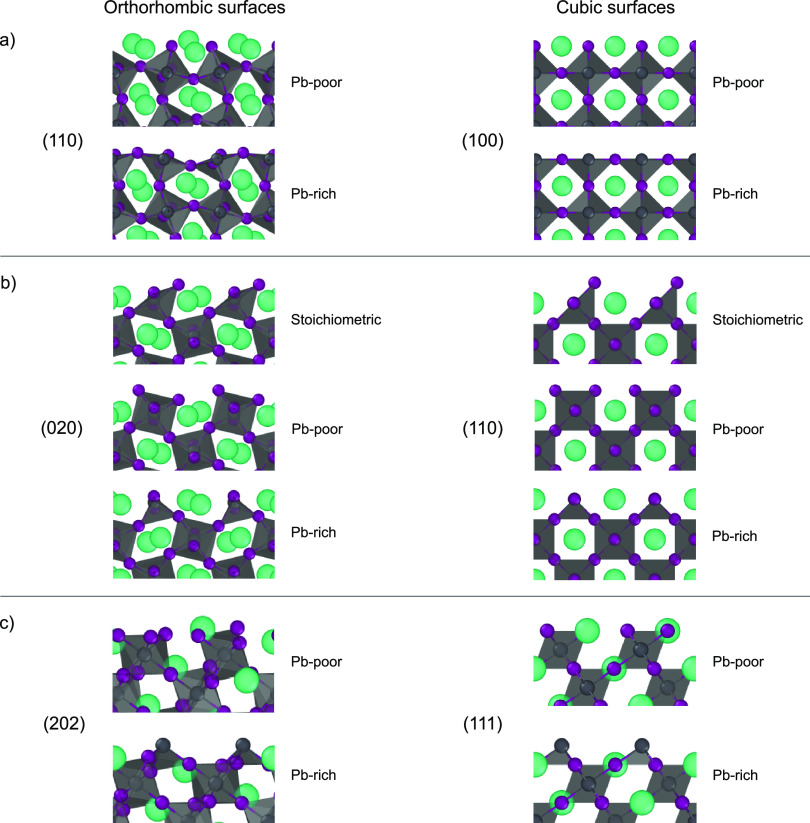
Structural models of the different surface orientations for orthorhombic CsPbI_3_ perovskite slabs. (a) (110)/(100), (b) (020)/(110), and (c) (202)/(111) surfaces are shown for the orthorhombic (left)/cubic (right) phases. For each of the slabs, the exposed surface is shown at the top.

To model the surfaces, we use slab models, shown in Figure 1a–c. These include CsPbI_3_ slabs with the (110), (020), and (202) planes of orthorhombic CsPbI_3_ exposed. Of these surfaces, the (110) and (020) planes of orthorhombic CsPbI_3_ are those most prevalent in XRD experiments.^37^ Despite the limited occurrence of the orthorhombic (202) plane in experiments, we include a slab with this orientation for completeness. This allows for the investigation of slabs with corners, edges, and faces of the PbI_*x*_ octahedra exposed, which is the case for the (110), (020), and (202) orthorhombic surfaces, respectively. Additionally, each surface is created with varying terminations. We differentiate between the following slab terminations: stoichiometric, Pb-poor, and Pb-rich; an explanation for these names can be found in section 1 of the Supporting Information. We emphasize that although here we refer to the slabs and their exposed planes in the orthorhombic form, all slabs attain time-averaged cubic structures at elevated temperatures because of thermal fluctuations. For comparison, the equivalent time-averaged cubic structure found when heating each orthorhombic surface is also shown in Figure 1. Additional details of the surface models can be found in section 1 of the Supporting Information.

A variety of CsPbI_3_ grain boundaries is studied to find their effects on the stability of halide perovskites. We focus on the 3Σ(112)(0.4, 0), 5Σ(210)(0.4, 0), and 3Σ(111)(0, 0) grain boundaries, which are created from the cubic phase of CsPbI_3_.^16^ We specifically investigate these grain boundary models because they do not contain unsaturated atoms with dangling bonds, the effects of which are probed with the above surface models. Additional details on the creation and naming of the grain boundaries can be found in section 2 of the Supporting Information.

### 2.2 Phase Stability near the Surface

**Figure 2:**
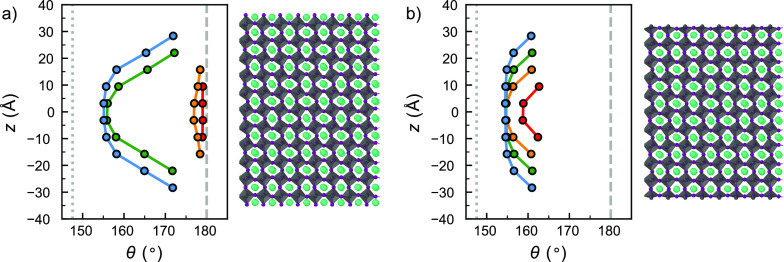
Time-averaged values of the Pb–I–Pb valence angle (*θ*) perpendicular to the surfaces grouped together along the depth of the slabs, with *z* = 0 indicating the center of the slab for the (a) Pb-poor and (b) Pb-rich terminations. The red, orange, green, and blue lines indicate the slabs of 4, 6, 8, and 10 octahedral cages thick, respectively. The time-averaged structures of the 10 layer slabs of CsPbI_3_ are shown next to the graphs. The values of the valence angle as obtained from density functional theory (DFT) calculations for the orthorhombic (148°) and cubic (180°) phases are indicated with the dotted and dashed lines, respectively.

To assess the effects of surfaces on the bulk structure, we simulate CsPbI_3_ slabs of the most commonly encountered (110) orthorhombic surface,^37^ which is equivalent to the (100) cubic surface.^38−40^ Slabs with a thickness of 4, 6, 8, or 10 octahedral cages and a Pb-poor or Pb-rich termination are simulated at a constant temperature of 300 K. We use the Pb–I–Pb valence angle (*θ*) to probe the effects of the surfaces on the bulk structure of the perovskite during the simulation. The time-averaged values of the Pb–I–Pb angles oriented in the direction perpendicular to the surfaces are shown in Figure 2a (Pb-poor) and Figure 2b (Pb-rich), together with the structural model of the slab with 10 layers. The structural models of slabs with a smaller thickness can be found in section 3 of the Supporting Information.

We find that regardless of the termination, the Pb–I–Pb valence angle near the center of the perovskite slab has on average a lower value (*θ*_MD_ = 155°) than that close to the surface (*θ*_MD_ = 160–175°). A comparison of these valence angles to those found for bulk structures using density functional theory (DFT) shows us that the center of the slab is orthorhombic (*θ*_DFT_ = 148°), while the regions near the surface tend to exhibit a more cubic structure (*θ*_DFT_ = 180°). We hypothesize that small perovskite domains attain a core–shell structure, in which the core has an orthorhombic structure and the surface layer is cubic-like, which we highlight could impact the optoelectronic properties of such domains.^41^ Notably, this agrees with experimental findings for CsPbI_3_, where progressively larger nanocrystals undergo a phase transition from a cubic-like phase to an orthorhombic phase.^42^ We find that this cubic-like surface layer is finite, approximately 2 nm thick, which might be a slight overestimation that stems from a small underestimation of the phase transition temperatures in ReaxFF.^33^ It has been shown that the large octahedral distortion in the orthorhombic phase of CsPbI_3_ results in poor Cs–I contacts in the material, which destabilizes the lattice, whereas the longer Cs–I contacts in cubic CsPbI_3_ result in a stabilization of the perovskite.^43^ Therefore, we propose that this core–shell structure could explain the enhanced stability of nanostructured CsPbI_3_ in experiments^42,44^ against the conversion into the nonperovskite yellow phase.^41,45^

### 2.3 Surface Stability

To investigate the thermal stability of perovskite surfaces, we subject a collection of perovskite slabs (Figure 1) to different temperatures ranging from 300 to 700 K in steps of 50 K. From these simulations, we determine the onset temperature of material degradation, which we use as an indication of the stability of the perovskite surfaces. In the definition of material degradation we include all processes that result in a crystal lattice that deviates from the pristine form, which include but are not limited to the formation of defect complexes, the clustering of atomic species, and the breakaway of atoms from the surface. The onset temperatures for the degradation of all slabs can be found in Table 1.

**Table 1:** Onset Temperatures for Lattice Degradation of Perovskite Slabs with Different Orientations and Terminations^a^

orientations	surface feature	stoichiometric	Pb-poor	Pb-rich
(110)	corner	–	*	550 K
(020)	edge	550 K	300 K	300 K
(202)	face	–	400 K	300 K

a For each surface, the octahedral feature that protrudes the surfaces is indicated. The (*) denotes a stable surface up to and including 700 K and (−) the absence of such a surface in the investigations.

We find that (110) orthorhombic slabs possess the highest resistance to thermal stress. The Pb-poor surface is stable for temperatures up to 700 K for simulations up to 5 ns, with the Pb-rich surface showing decomposition of the lattice from 550 K and higher. This stability matches the trend in surface formation energies observed for the equivalent cubic surfaces in the ReaxFF force field validation (see section 4 of the Supporting Information). A lower thermal stability is observed for the (020) orthorhombic surface. The stoichiometric (020) surface of CsPbI_3_ is stable up to 450 K with the rearrangement of surface iodine atoms occurring at 500 K (details in section 5 of the Supporting Information) and degradation of the surface occurring at 550 K and higher. Both the Pb-rich and Pb-poor terminations of the (020) orthorhombic surface are unstable, exhibiting the clustering of atomic species near the surface at 300 K. Finally, the (202) orthorhombic surface is the least stable, with the Pb-poor termination degrading from 400 K and up and the Pb-rich termination already decomposing at 300 K. On the basis of these results, we rank the surface orientations from most stable to least stable as (110) > (020) > (202). We emphasize that this observed stability trend correlates well with the occurrence of these surfaces in XRD experiments, in which the most stable surfaces appear predominantly.^37−40^

**Figure 3:**
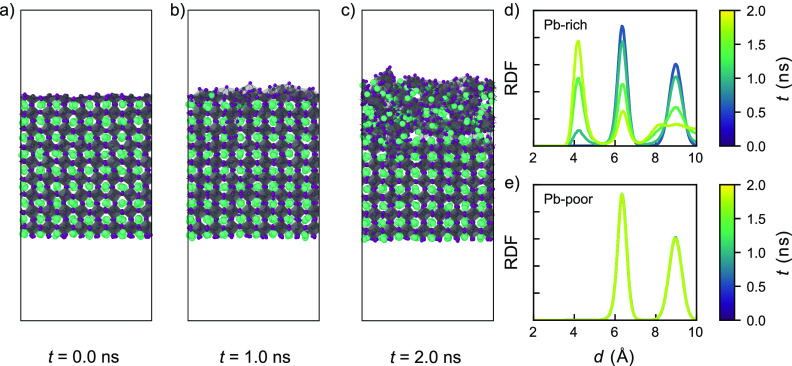
Degradation of a (110) orthorhombic perovskite surface at 600 K, with the Pb-rich surface on the top and the Pb-poor surface at the bottom of the slab. (a–c) Structural snapshots of the degrading perovskite slab. (d, e) Time evolution of the Pb–Pb radial distribution function (RDF) for the Pb-rich and Pb-poor surfaces of the perovskite slab, demonstrating that the degradation of the Pb-rich surface starts from a clustering of Pb species.

For more insights into the degradation dynamics, we take a closer look at the degradation of a (110) orthorhombic slab simulated at 600 K. In Figure 3a–c we show snapshots of the lattice degradation during the simulation. Consistent with our earlier observations, the snapshots show that under these conditions the (110) Pb-poor surface remains intact, while the Pb-rich surface shows decomposition, leading to the formation of a Pb_*x*_I_*y*_ complex. To quantify the degradation processes, we analyze the radial distribution functions (RDFs) between the Pb–Pb atom pairs in the simulated system in Figure 3d and e and for the remaining atom pairs in section 6 of the Supporting Information. On the Pb-rich surface, the Pb–Pb RDF shows a simultaneous increase in the peak at 4.2 Å and a decrease in the peak at 6.3 Å, the timing of which coincides with the onset of degradation seen in Figure 3a–c. On the contrary, the Pb-poor surface does not show any change in peak intensity and therefore shows no signs of decomposition. On the basis of these observations, we conclude that the degradation of the perovskite lattice can be characterized by the clustering of Pb atoms, which has been observed previously in experiments at perovskite grain boundaries.^27^ By comparing the position of the emerging Pb–Pb peak (4.2 Å) to the interatomic distances of Pb species from DFT in layered PbI_2_ (4.55 Å) or yellow phase CsPbI_3_ (4.65 Å), we can conclude that the decomposition product is not a pure form of either of these two material phases but a more amorphous Pb_*x*_I_*y*_ domain. The formation of these domains matches observations from several experimental reports in which the formation of PbI_2_ domains is observed in electron microscopy experiments at grain boundaries.^27−29^

**Figure 4:**
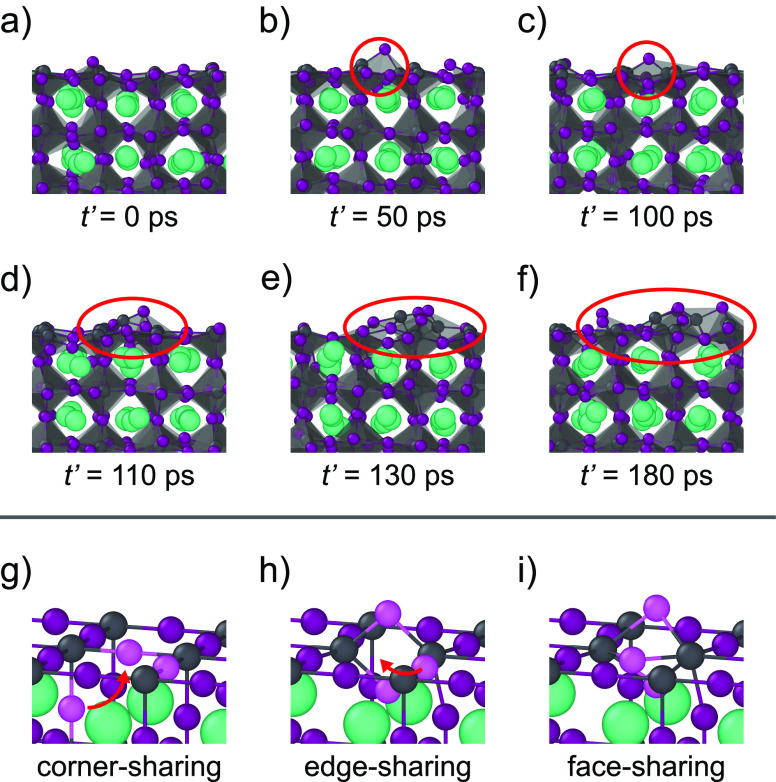
Snapshots of the degradation of a Pb-rich (110) orthorhombic perovskite surface. (a) Nondegraded perovskite surface. (b, c) A relatively long-lived iodine Frenkel defect at the perovskite surface, resulting in an edge-sharing complex. (d, e) A face-sharing complex at the surface that starts to break away from the surface. (f) Decomposed perovskite lattice at the surface. (g–i) Schematic representation of the critical steps in the degradation mechanism where neighboring PbI_*x*_ octahedra change from corner- to edge- to face-sharing. The species highlighted in pink are the I atoms involved in the formation of the surface defect. The figure makes use of a shifted time axis, with *t*′ = 0 ps corresponding to *t* = 0.75 ns in simulation time.

From all the decomposing CsPbI_3_ surfaces reported above, we observe that all surfaces degrade through a similar mechanism. To illustrate the key steps of this mechanism, we show the degradation of the Pb-rich orthorhombic (110) surface at 600 K as an example in Figure 4. In the first step of the degradation process, an iodine Frenkel defect is formed in the perovskite lattice (Figure 4a–c). As a result of this, two PbI_*x*_ octahedra form an edge-sharing complex, as opposed to the corner-sharing geometry in a regular perovskite lattice. Here, two Pb atoms are bound together by two I atoms. This edge-sharing complex acts as a metastable state in our simulations, exhibiting lifetimes of up to 50 ps. In the next step, an additional I atom is added to the edge-sharing complex, transforming it into a face-sharing complex, in which the interatomic distance of the Pb atoms involved in the complex significantly decreases (Figure 4d). We regard the formation of this face-sharing complex as the starting point of the degradation of the surface. Shortly after its formation, this face-sharing complex breaks away from the surface, initiating the decomposition of the perovskite lattice near the surface (Figure 4e and f). Altogether we find that temperature affects the rate at which the degradation reaction proceeds, with more significant thermal fluctuations at elevated temperatures resulting in a faster degradation of the perovskite lattice.

We highlight that the critical steps of the degradation mechanism of perovskite surfaces (Figure 4g–i) resemble the degradation near iodine vacancies in the bulk of CsPbI_3_.^33^ In this previous study, we also found that iodine interstitials do not initiate the degradation of perovskites. Thus, we establish that Frenkel defects, which previously have been connected mainly to ion migration,^21,46^ are a main cause of lattice instabilities that originate from the vacancy part of the interstitial–vacancy defect pair. Based on the above, we note that two distinct features make perovskite surfaces more prone to degradation: (1) an abundance of dangling bonds and (2) a lack of steric hindrance. Using DFT calculations, it has been shown that the density and type of dangling bonds affect the formation energies of perovskite surfaces.^47^ Here, we posit that such dangling bonds impact not only the thermodynamic stability of these surfaces but also the dynamical stability. In particular, we find that undercoordinated Pb species readily form new bonds that then progressively degrade the halide perovskite. Additionally, the stability trend of the perovskite surfaces found earlier can be classified according to the protruding surface features as corner > edge > face. We stress that in this ranking the PbI_*x*_ octahedra, and specifically the I species, experience an increasingly smaller steric hindrance from the Cs species at the surface, allowing for the movement of these octahedra and thus a larger tendency for perovskite degradation. Finally, these two factors also explain the high thermal stability of the Pb-poor (110) orthorhombic surface. In addition to the absence of Pb dangling bonds, the Cs atoms also sterically hinder the movement of the undercoordinated I species at the surface. Together, this inhibits the decomposition, making the surface very stable.

### 2.4 Effect of Additional Point Defects on Surfaces

To assess the effects of additional point defects on the stability of CsPbI_3_ perovskite surfaces, we use the Pb-poor (110) orthorhombic surface as a model system because this perovskite surface has shown high thermal stability (section 2.3). Because the surface is dominated by Cs and I species, with theoretical support for the occurrence of vacancies of both species,^48^ we look at the effect of such vacancies at 600 K, individually and when they are clustered. In the remainder of this work, we refer to the Cs and I vacancies as V_Cs_ and V_I_. The structural models used for the investigation of these defects can be found in section 7 of the Supporting Information.

**Figure 5:**
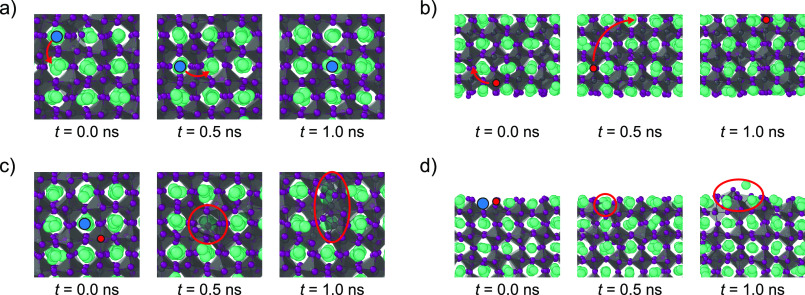
Snapshots of different point defects in CsPbI_3_ slabs. (a) Top view of a surface with a V_Cs_ defect, showing the motion of the defect across the surface. (b) Side view of a surface with a V_I_ defect, showing the migration of the defect away from the surface into the bulk. (c, d) A top and side view of a perovskite surface with a V_Cs_ and V_I_ defect pair, showing the degradation of the perovskite surface caused by the presence of this defect at the surface. The V_Cs_ and V_I_ defects are indicated with a blue and red sphere, respectively.

In their isolated form, the defects have relatively benign effects on the lattice stability, which appears very similar to that observed for bulk CsPbI_3_,^33^ but when the defects form pairs, it is found that they do significantly impact the stability of the perovskite lattice. The V_Cs_ defect remains on the surface of the slab, where it migrates across the surface as shown in Figure 5a, without resulting in the degradation of the lattice. In contrast, the motion of V_I_ is not bound to the surface of the perovskite. As shown in Figure 5b, a V_I_ defect can migrate into and out of the bulk of the perovskite. Moreover, in some cases the V_I_ defect causes the perovskite surface to degrade; however, this process is not restricted to perovskite surfaces and also occurs in the bulk of inorganic perovskites.^33^ In Figure 5c and d, the effects of a closely spaced defect pair of V_I_ and V_Cs_ are shown. During the simulation, we observe that this defect cluster tends to stay together at a fixed position on the surface, only occasionally splitting into isolated V_Cs_ and V_I_ defects, which indicates an enhancement of the defect-trapping ability of surfaces for halide defects,^49^ specifically when paired with cation vacancies. When the defect pair remains clustered, the pair is particularly detrimental for the stability of the perovskite lattice. Specifically, we observe Pb species readily moving away from their original position in the lattice in close proximity to the defect pair (*t* = 0.5 ns in Figure 5), initiating the degradation of the lattice by allowing Pb and I species to cluster and form an amorphous Pb_*x*_I_*y*_ domain. We connect the tendency for a defect pair to act as a degradation center to the earlier-established factors for perovskite degradation: the presence of Pb dangling bonds and limited steric hindrance for I species near the defect pair.

### 2.5 Grain Boundaries

**Figure 6:**
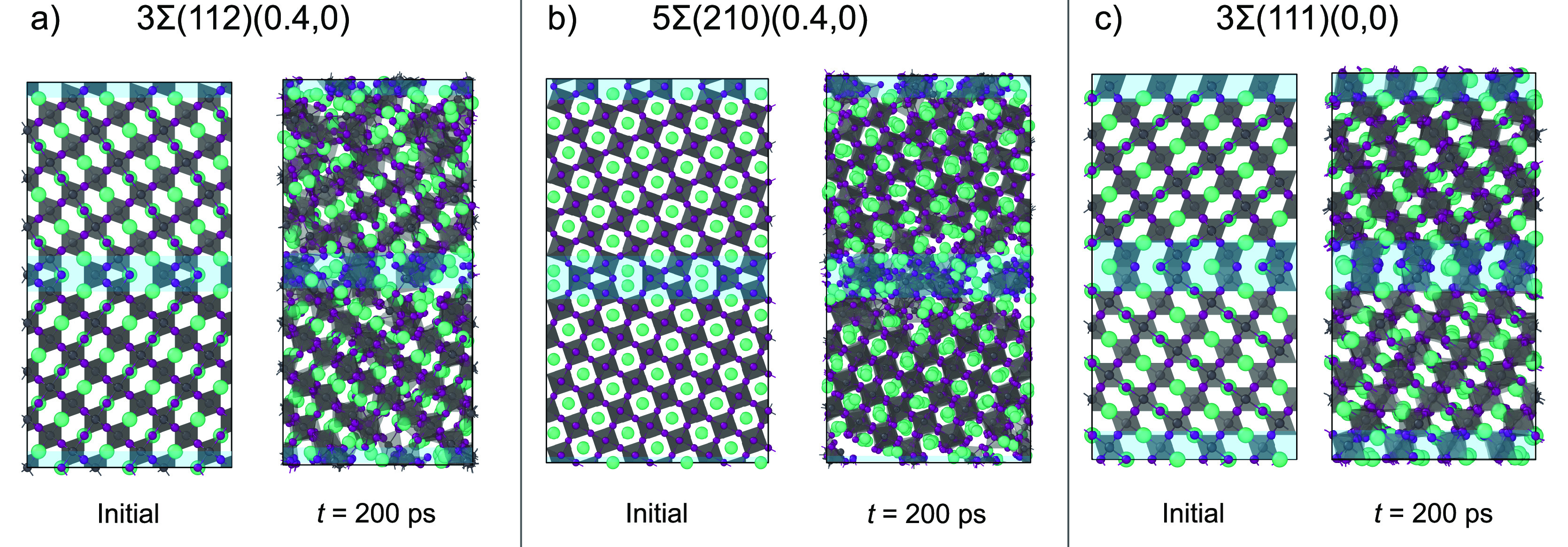
Dynamical evolution of the CsPbI_3_ grain boundaries at 600 K after 200 ps from their initial structure. (a) 3Σ(112)(0.4, 0) grain boundary. (b) 5Σ(210)(0.4, 0) grain boundary. (c) 3Σ(111)(0, 0) grain boundary. The blue areas highlight the grain boundaries in the structures.

We assess the stability of grain boundaries from simulations at 600 K. The structure of the model systems after 200 ps of simulation is shown in Figure 6. The 3Σ(112)(0.4, 0) (Figure 6a) and 5Σ(210)(0.4, 0) (Figure 6b) grain boundaries exhibit clustering of atomic species in the grain boundary region. This clustering results in the formation of amorphous Pb_*x*_I_*y*_ domains, an observation that is consistent with the surfaces presented above (section 2.3) and the experimentally observed degradation of perovskites at grain boundaries.^27−29^ Contrary to the other two grain boundaries, the 3Σ(111)(0, 0) grain boundary (Figure 6c), also known as a twinning plane,^18^ does not show any degradation throughout the duration of the simulation (2 ns).

**Figure 7:**
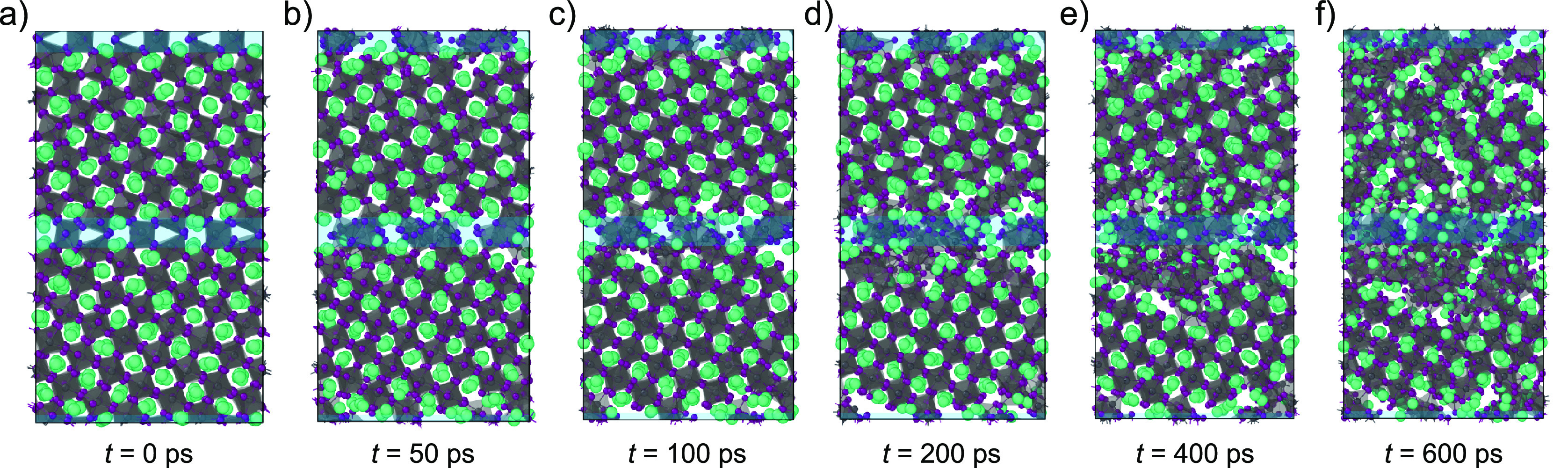
Degradation of a 5Σ(210)(0.4, 0) grain boundary. (a–c) The degradation initiates at the grain boundary and (d–f) proceeds into the bulk of the perovskite. The blue areas in the figures highlight the grain boundary.

To investigate the degradation mechanism of these grain boundaries in more detail, we look into the time evolution of the degradation 5Σ(210)(0.4, 0) grain boundary (Figure 7), which for the 3Σ(112)(0.4, 0) and 3Σ(111)(0, 0) grain boundaries are shown in section 8 of the Supporting Information. Careful inspections point to a general mechanism in which the degradation is initiated by the movement of iodine atoms near the grain boundary, resulting in the formation of small Pb_*x*_I_*y*_ domains at the grain boundary (Figure 7a and b). Similar to perovskite surfaces, we find that these small Pb_*x*_I_*y*_ domains grow progressively larger (Figure 7c), resulting in the degradation progressing into the perovskite bulk (Figure 7d–f). Owing to an absence of any dangling bonds in the grain boundary models, we can connect this observed material instability to the lack of steric hindrance at grain boundaries. In particular, both the 3Σ(112)(0.4, 0) and 5Σ(210)(0.4, 0) grain boundaries lack Cs species that can block the clustering of closely spaced Pb and I species, making them unstable. In contrast, the grain boundary 3Σ(111)(0, 0) has intact face-sharing PbI_*x*_ octahedra at the grain boundary with cavity-filling Cs species that sterically hinder the movement of these octahedra. Altogether this stabilizes the 3Σ(111)(0, 0) grain boundary, making this grain boundary more stable than its (111) cubic surface analogue, which, based on its equivalent (202) orthorhombic surface, is unstable from 400 K onward.

## 3 Conclusion

In summary, using a ReaxFF force field, we study structural and thermal stability effects of surfaces and grain boundaries in the inorganic halide perovskite CsPbI_3_. We show that surfaces affect the crystal phase close to the surface, which attains a cubic-like structure that is approximately 2 nm in size. We believe that this surface region is responsible for the enhanced structural stability of nanostructured CsPbI_3_. Under a thermal stress that ranges from 300 to 700 K, we find a stability trend for orthorhombic CsPbI_3_ of (110) > (020) > (202). This trend matches the occurrence of these surfaces in experiments, in which the most stable surfaces are most predominantly observed. Comparing all investigated structures, we propose two important factors that are responsible for the degradation of perovskites: (1) the presence of dangling bonds, particularly for Pb species, and (2) a lack of steric hindrance, especially for I species. These two factors explain the high thermal stability observed for the Pb-poor (110) orthorhombic surface. The surface only has I dangling bonds and no Pb dangling bonds, and the motion of the I species is blocked by the Cs species on the surface, resulting in a stable surface. All other surfaces that do not satisfy these two conditions eventually decompose through a common mechanism. In this mechanism first an iodine Frenkel defect is formed in close proximity to the surface; after some time this defect grows to form a complex of two face-sharing PbI_*x*_ octahedra; and finally the complex breaks away from the surface, growing larger by accumulating more Pb and I species, leading to the formation of a large amorphous Pb_*x*_I_*y*_ domain on the surface.

The stability of aforementioned surfaces deteriorates when additional point defects (V_I_ and V_Cs_) are created on the surfaces, which results in the formation of Pb dangling bonds and a local decrease of steric hindrance. The latter factor also explains the stability of the investigated grain boundaries. For example, although the 3Σ(112)(0.4, 0) and 5Σ(210)(0.4, 0) grain boundaries do not contain any dangling bonds, the lack of steric hindrance for Pb and I species facilitates the clustering of these species, leading to the degradation of the material. On the contrary, the 3Σ(111)(0, 0) grain boundary contains cavity-filling Cs species that sterically hinder the typical degradation-inducing movement of the face-sharing octahedra, making it stable up to 600 K.

Based on the above, we propose that strategies to stabilize halide perovskites can include the following aspects: (i) passivating defects, primarily those leading to unsaturated bonds, i.e., halide vacancies, through passivating agents such as halogens like F^50^ and Cl^51^ or carbonyl-^52^ and azo-^53^ containing ligands; (ii) the grafting of surfaces with sterically hindering groups, such as phenylalkylammonium^54^ and even bulkier organic groups;^55^ (iii) optimizing the synthesis conditions, through the type of precursors, solvents, additives, etc., to stimulate the growth of nondetrimental surface orientations,^56^ reduce the formation of grain boundaries,^57^ and suppress the formation of defects altogether.^58^

## 4 Computational Details

### 4.1 Molecular Dynamics

ReaxFF molecular dynamics simulations were performed in AMS2021.^59^ All ReaxFF simulations were done using the earlier developed CsPbI_3_ ReaxFF force field;^33^ a validation of this force field for CsPbI_3_ surfaces can be found in section 4 of the Supporting Information. Before the molecular dynamics runs, all structural models were optimized with the ReaxFF force field. The dynamical simulations used a simulation time step of 0.25 fs. The thermostat and barostat use a damping constant of τ_T_ = 100 fs and τ_p_ = 2500 fs, respectively. Whenever slabs were simulated, the vacuum layer used was at least 50 Å in the *z*-direction. In the case of slabs, the barostat was only allowed to scale the nonvacuum directions of the model system (*x*- and *y*-directions). The initial velocities of the particles were assigned according to a Maxwell–Boltzmann distribution of the initial temperature. Simulation snapshots and structural models were all visualized using OVITO.^60^

In the simulations investigating the structural effects of surfaces on the bulk structure of perovskites, we equilibrated the systems to the target temperature of 300 K in an NPT ensemble for 200 ps. During the equilibration stage, we employed a Berendsen thermostat and Berendsen barostat^61^ to control the temperature and pressure, respectively. Production runs were started from the final frame of the equilibration run and took 200 ps. In the production runs, the temperature and pressure were controlled with a NHC thermostat^62^ with a chain length of 10 and an MTK barostat.^63^ The time-averaged values of the Pb–I–Pb valence angles were extracted from the time-averaged structure we obtained by averaging the atomic positions over the full duration of the 200 ps production simulations.

The stability of the perovskite surfaces and grain boundaries was investigated using a three-step approach. In the first two steps, the equilibration stage, we used a Berendsen thermostat and Berendsen barostat to control the temperature and pressure. The first step was used to slowly heat the system from 300 K to the desired target temperature during 100 ps, with the second step maintaining the system at its constant target temperature for 100 ps. The full equilibration of the system was run in the nonreactive mode of ReaxFF in AMS2021, in which the bonds can only be updated but not newly formed, to prevent unwanted reactions during the equilibration of the system. The final stage, the production simulation, was run in an NPT ensemble for which the starting point was the final frame of the equilibration. The temperature and pressure were controlled, respectively, with an NHC thermostat with a chain length of 10 and an MTK barostat. Each of the production runs was 2 ns long, except in the assessment of the stability of the Pb-poor (110) orthorhombic surface for which we used 5 ns long simulations.

### 4.2 Density Functional Theory

Density functional theory (DFT) calculations were performed with the projector augmented wave (PAW) method as implemented in the Vienna Ab-Initio Simulation Package (VASP).^64−67^ The electron exchange-correlation interaction was described using the Perdew, Burke, and Ernzerhof (PBE) functional^68^ with long-range dispersive interactions accounted for by the DFT-D3(BJ) dispersion correction.^69^ We treated the outermost electrons of Cs (5s^2^5p^6^6s^1^), Pb (5d^10^6s^2^6p^2^), and I (5s^2^5p^5^) as valence electrons with the plane wave basis set expanded to an energy cutoff of 500 eV.

The geometries were optimized by allowing all the ionic positions, cell shape, and cell volume to change until convergence of 1 × 10^–3^ meV and 10 meV Å^–1^ was reached in energy and forces, respectively. The Brillouin zones were sampled using a Monkhorst–Pack mesh,^70^ with the following *k*-space grids resulting in energy convergence to within 1 meV/atom: CsI: 12 × 12 × 12; PbI_2_: 11 × 11 × 7; cubic CsPbI_3_: 10 × 10 × 10; orthorhombic CsPbI_3_: 7 × 7 × 5; yellow phase CsPbI_3_: 13 × 6 × 4.

## Supporting Information Available

Structural models of the surfaces and grain boundaries; time-averaged structures of four, six, and eight octahedral layer thick perovskite slabs; ReaxFF force field validation using surface energies; illustration of the rearrangement of surface iodine for the stoichiometric (020) orthorhombic surface; explanation of RDFs and overview of the RDFs for remaining atom pairs; structural models of the defective surfaces; snapshots of the evolution of the 3Σ(112)(0.4, 0) and 3Σ(111)(0, 0) grain boundaries (PDF)
Structure files of the bulk perovskite and precursor structures, the pristine and defective perovskite slabs, and the perovskite grain boundaries (ZIP)
Simulation trajectories at 600 K of the (110) orthorhombic perovskite slab during degradation, (110) orthorhombic Pb-poor perovskite slabs with isolated and paired V_Cs_ and V_I_ defects, and the time evolution of the 3Σ(112)(0.4, 0), 5Σ(210)(0.4, 0), and 3Σ(111)(0, 0) perovskite grain boundaries (ZIP)
