# Supplementary material for: What Happens at Surfaces and Grain Boundaries of Halide Perovskites: Insights from Reactive Molecular Dynamics Simulations of CsPbI3
Source: ACS Appl Mater Interfaces. 2022 Aug 30;14(36):40841–50. doi: 10.1021/acsami.2c09239 (PMC9478958; doi:10.1021/acsami.2c09239)
Supplement: Supplementary file 1 — am2c09239_si_001.pdf [file am2c09239_si_001.pdf]

Supporting Information: What Happens at Surfaces and Grain Boundaries of Halide Perovskites: Insights from Reactive Molecular Dynamics Simulations of $CsPbI_3$

Mike Pols,[†,‡,‖] Tobias Hilpert,[†,¶,‖] Ivo A.W. Filot,[¶,‡] Adri C.T. van Duin,[§] Sofía Calero,[*,†] and Shuxia Tao[*,†,‡]

†*Materials Simulation & Modelling, Department of Applied Physics, Eindhoven University of Technology, 5600 MB, Eindhoven, The Netherlands*

‡*Center for Computational Energy Research, Department of Applied Physics, Eindhoven University of Technology, 5600 MB, Eindhoven, The Netherlands*

¶*Laboratory of Inorganic Materials Chemistry, Schuit Institute of Catalysis, Department of Chemical Engineering and Chemistry, Eindhoven University of Technology, 5600 MB, Eindhoven, The Netherlands*

§*Department of Mechanical Engineering, Pennsylvania State University, University Park, PA 16802, United States*

‖*Contributed equally to this work*

E-mail: s.calero@tue.nl; s.x.tao@tue.nl

Contents

Supporting Notes **S3**

1 Structural models for surfaces S3

1.1 Surface termination names S3

1.2 Surface models S4

2 Structural models for grain boundaries S5

2.1 Grain boundary names S5

2.2 Grain boundary models S6

3 Structural effects of surfaces S7

4 Force field validation S8

5 Surface rearrangements of iodine S10

6 Radial distribution functions S11

7 Defective surfaces S13

8 Grain boundary evolution S14

References **S15**

Supporting Notes

1 Structural models for surfaces

1.1 Surface termination names

The different surface terminations are named according to the relative concentration of species at these surfaces. To determine the surface concentration of the species, we create perovskite slabs with the same termination at both ends and consequently calculate the ratios of the atoms that make up these slabs. An overview of the ratios found for the different slabs is shown in Table S1. The naming scheme is based on the relative ratio of the I and Pb species at each surface, resulting in the following names: stoichiometric (I:Pb = 3), Pb-poor (I:Pb > 3) and Pb-rich (I:Pb < 3).

Table S1: Relative ratio of the occurrence of atomic species as determined for perovskite slabs with the same termination at both sides of the perovskite slab.

Orientation	Termination	Pb:Cs	I:Cs	I:Pb
(110)	Pb-poor	0.89	2.78	**3.13**
	Pb-rich	1.13	3.25	**2.89**
(020)	Stoichiometric	1.00	3.00	**3.00**
	Pb-poor	1.00	3.15	**3.15**
	Pb-rich	1.00	2.86	**2.86**
(202)	Pb-poor	0.94	3.00	**3.20**
	Pb-rich	1.07	3.00	**2.81**

1.2 Surface models

In this work the surface models are created in AMS2021.[1] An orthorhombic unit cell is used as the starting point for the surface models. Each model slab is at least 50 Å thick, with the lateral dimension spanning at least 45 Å. An overview of the full structural models of the doubly terminated (Pb-poor and Pb-rich) slabs is shown in Figure S1.

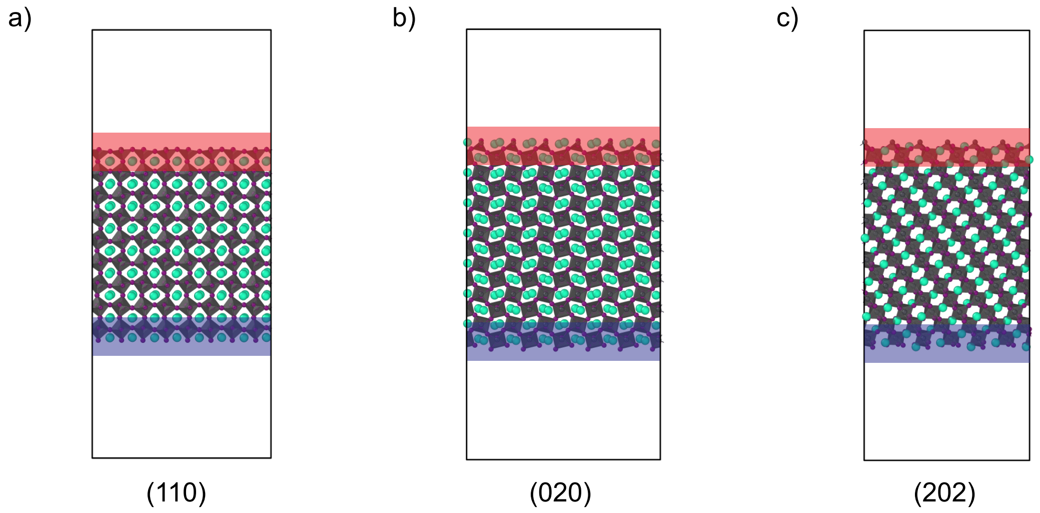

Figure S1: Structural models of the Pb-poor surfaces as created from orthorhombic $CsPbI_3$. (a) (110) orthorhombic surface. (b) (020) orthorhombic surface. (c) (202) orthorhombic surface. The Pb-rich and Pb-poor terminated surfaces are indicated with the red and blue color, respectively.

2 Structural models for grain boundaries

2.1 Grain boundary names

The names of the grain boundaries follow from the work of Guo et al.[2] The grain boundaries in this work are created with the coincidence site lattice (CSL) model. In this approach, two grains are tilted with a certain angle, until their surface planes coincide. An example of such a grain boundary structure is the $3\Sigma(112)(0.4, 0)$ grain boundary. In this naming scheme the 3 in front of Σ denotes that every third point on the surface planes coincides. The number that follows Σ, (112), denotes the lattice plane along which the intersection occurs. The final two numbers indicate the overall fractional shift of the grains with respect to each other. For $(0.4, 0)$ this means that one of the grains is shifted along the [100] direction by 40% of the length of the unit cell vector and without any shift along the [010] direction. Note these shift directions hold because the grain boundaries are formed perpendicular to the z-axis.

2.2 Grain boundary models

The grain boundary models in this work are created with the `aimsgb` package.[3] A cubic $CsPbI_3$ unit cell is used as the starting point for the grain boundary models. An overview of the initial structures of the grain boundary models is shown in Figure S2. In each model the grain boundaries are separated by at least 25 Å. The lateral dimensions of the grain boundary models are at least 30 Å in size.

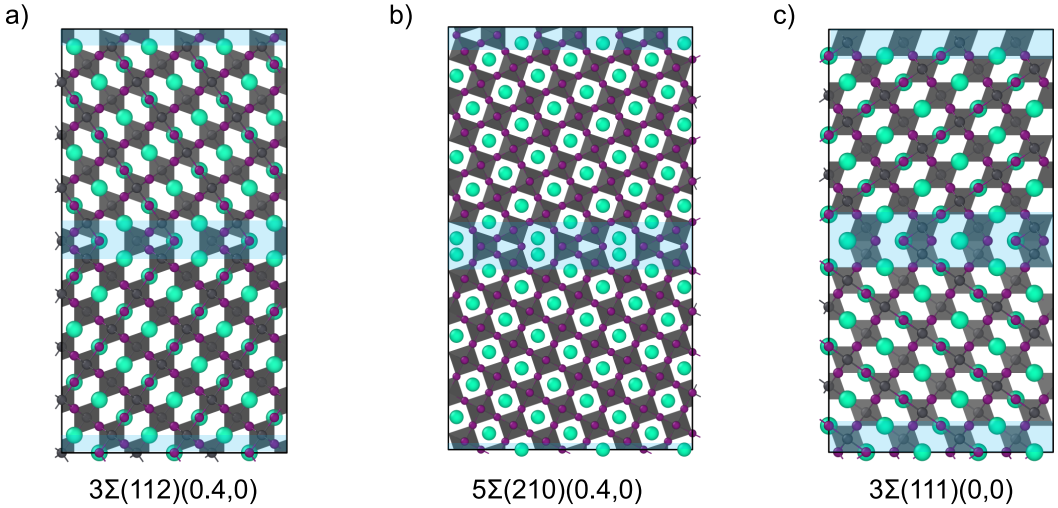

Figure S2: Structural models of the grain boundaries as created from cubic $CsPbI_3$. (a) $3\Sigma(112)(0.4, 0)$ grain boundary. (b) $5\Sigma(210)(0.4, 0)$ grain boundary. (c) $3\Sigma(111)(0, 0)$ grain boundary. The blue areas highlight the grain boundaries in the structures.

3 Structural effects of surfaces

The time-averaged structures of the 10 layer thick slabs of $CsPbI_3$ are shown in Figure 2 in the manuscript. For a complete overview of the slabs with varying thickness, the time-averaged structural models of the slabs of 4, 6 and 8 octahedral cages thick are shown in Figure S3, which demonstrates that thinner slabs attain more cubic-like structures.

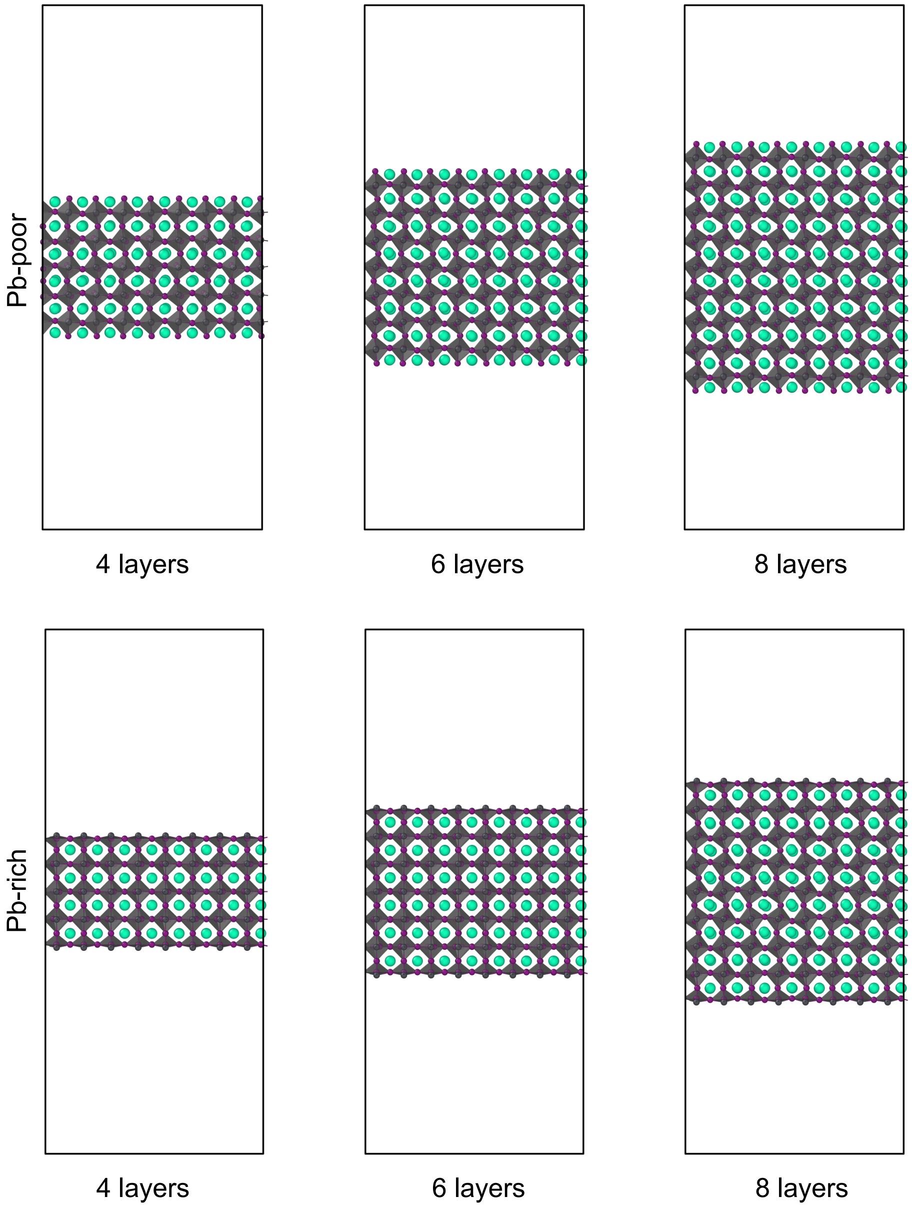

Figure S3: Time-averaged perovskite slab structures for both Pb-poor and Pb-rich slabs of 4, 6 and 8 layers of octahedral cages.

4 Force field validation

Slab geometries were created from optimized bulk structures of the cubic phase of $CsPbI_3$ in both DFT and ReaxFF. During relaxation of the slabs, only the ionic positions were allowed to change until convergence was obtained in energy and forces. In the DFT calculations a $6 \times 6 \times 1$ k-points mesh was used during slab optimizations. At least 15 Å of vacuum was included to minimize the interaction between periodic images of the slab. The structural models of the perovskite slabs are shown in Figure S4.

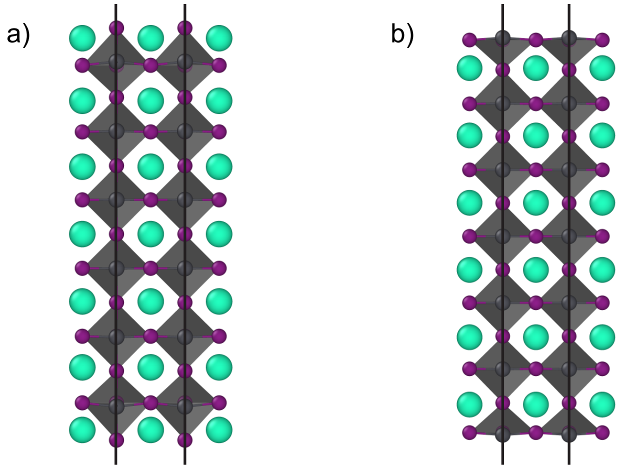

Figure S4: Structural models of the slabs used in the validation of the force field. (a) Pb-poor terminated slab. (b) Pb-rich terminated slab. The simulation cell is indicated with the black lines.

Surface energies (γ) were calculated as

$$\gamma = \frac{E_{\text{slab}} - \left(n \cdot E_{\text{CsPbI}_3} \pm E_{\text{precursor}}\right)}{2A} \tag{S1}$$

where E_{slab} is the energy of the perovskite slab, n the number of formal units of $CsPbI_3$, E_{CsPbI_3} is the energy of cubic $CsPbI_3$, $E_{\text{precursor}}$ is the energy of the perovskite precursor and A the surface area of the perovskite slab. To make use of a common point of reference for the validation, we pick CsI as the precursor, resulting in $n = 6$ and an addition of the precursor energy ($+E_{\text{precursor}}$) for the Pb-poor terminated slab and $n = 7$ and a subtraction of the precursor energy ($-E_{\text{precursor}}$) for the Pb-rich terminated slab.

An overview of the surface areas and calculated surface energies of the perovskite slabs with the Pb-rich and Pb-poor termination is shown in Table S2. From this comparison we find that the used ReaxFF force field matches the stability trend of our DFT simulations, as well as those found in literature.[4,5] In both computational methods the Pb-poor surface termination is found to be significantly more stable than the Pb-rich surface termination. Although the relative magnitude for DFT and ReaxFF does not exactly match, the comparison validates that the force field is transferable for the simulation of surfaces.

Table S2: The areas (A) and surface energies (γ) for the different $CsPbI_3$ slab terminations as calculated with DFT and ReaxFF.

Surface termination	A ($\mathrm{\AA}^2$)	γ_{DFT} ($\mathrm{J\,m^{-2}}$)	γ_{ReaxFF} ($\mathrm{J\,m^{-2}}$)
Pb-poor	39.58	0.095	-0.087
Pb-rich	39.58	0.222	0.144

5 Surface rearrangements of iodine

For the stoichiometric orthorhombic (020) surface it was observed that iodine species would migrate across the surface from 500 K and up. To illustrate this behavior, some simulation snapshots from 500 K simulations are shown in Figure S5. We underline that the jump of an iodine atom to a neighboring Pb atom (Figure S5a) forms local Pb-rich and Pb-poor octahedra, which become the degradation centers at elevated temperatures.

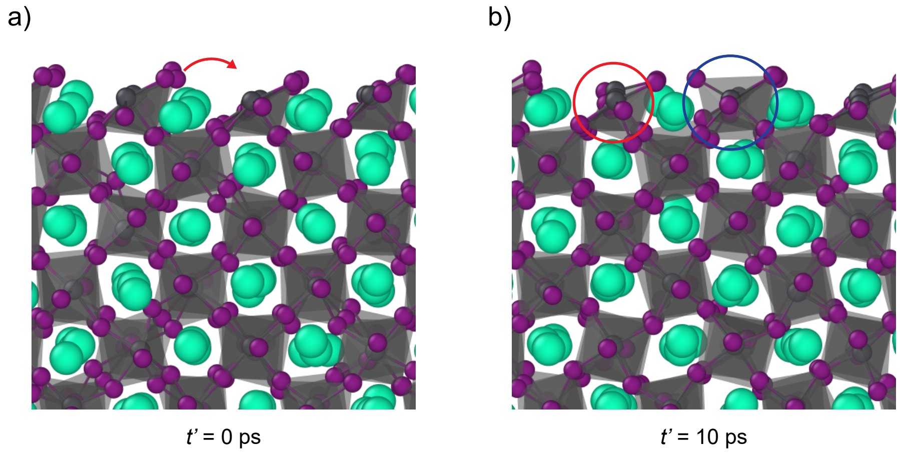

Figure S5: Snapshots of the rearrangement of surface iodine atoms for the orthorhombic (020) stoichiometric surface at 500 K. (a) Stuctural arrangement before the migration of surface iodine. (b) Surface geometry after surface iodine rearrangment, with the blue and red circle indicating a Pb-poor and Pb-rich octahedra, respectively. A shifted time axis t' is used.

6 Radial distribution functions

The RDF between the atoms of the species a and b is calculated using

$$g_{ab}\left(r\right) = \frac{1}{N_a N_b} \sum_{i=1}^{N_a} \sum_{j=1}^{N_b} \langle \delta\left(\left|\vec{r}_i - \vec{r}_j\right| - r\right)\rangle, \tag{S2}$$

which sums over Dirac delta functions δ that are offset by the interatomic distance between the ith atom of species a located at $\vec{r}_i$ and the jth atom of species b located at $\vec{r}_j$. The quantity is averaged over the full length of the simulation indicated by the ensemble averaging ($\langle \cdots \rangle$).

In this work, we employ the radial distribution function (RDF) as implemented in the `MDAnalysis` package.[6,7] Surface specific RDFs are obtained by only including those atoms that are within 4 layers of Pb atoms from the surface. The time information is obtained by dividing the trajectory of the degradation simulation into five blocks of equal length, each corresponding to 400 ps of data. Subsequently, the RDF is determined for each of these spatial and temporal blocks, resulting in RDFs with space- and time-resolved information on the degradation in our system. A demonstration of the partitioning into surface-specific regions and the remaining RDFs not treated in the main text (atom pairs: Pb-Pb, Pb-Cs, Pb-I, Cs-Cs, Cs-I and I-I) of the Pb-rich and Pb-poor (110) orthorhombic surface of $CsPbI_3$ are shown in Figure S6.

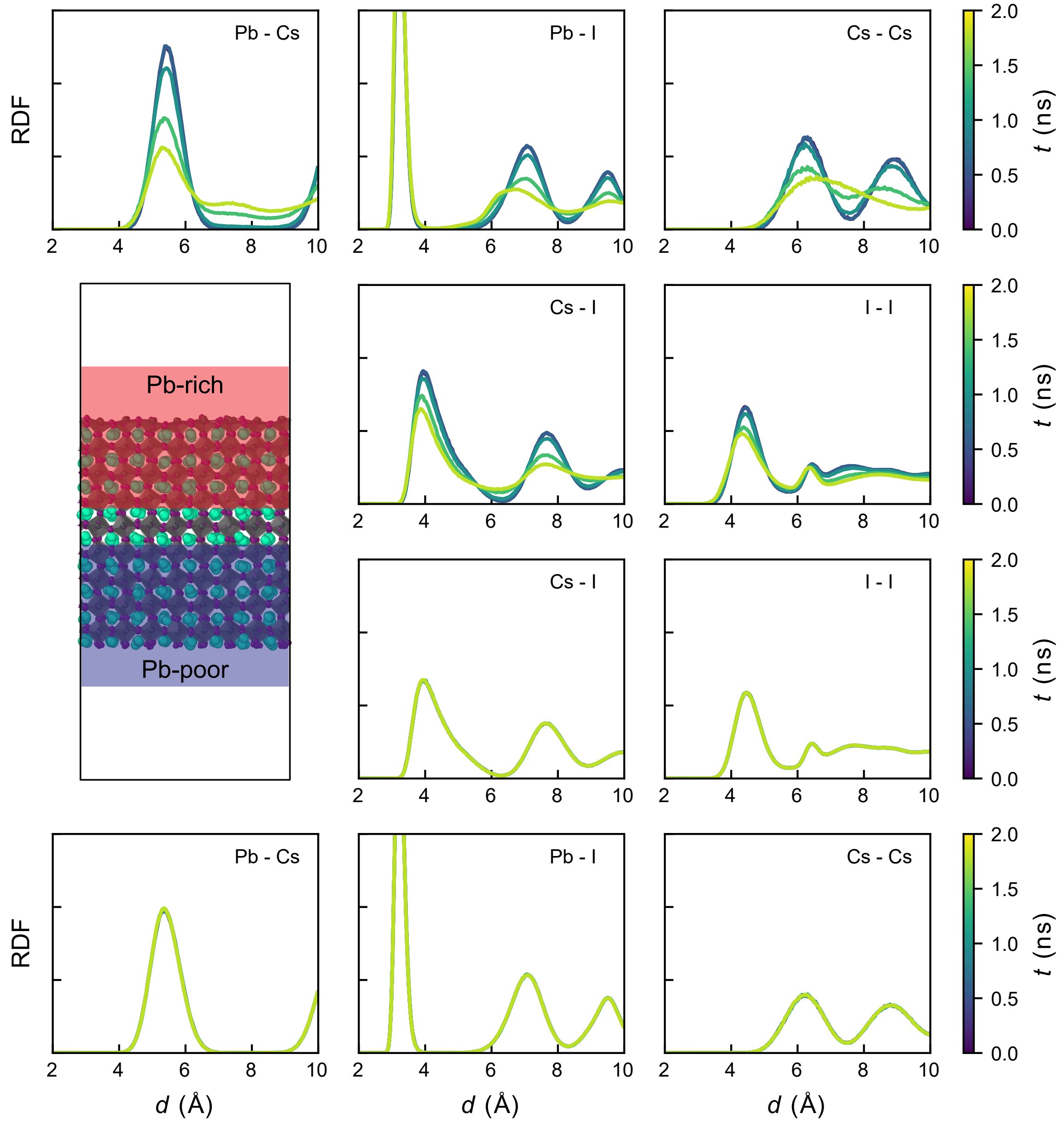

Figure S6: Time-resolved radial distribution functions (RDFs) for the (110) surface of orthorhombic $CsPbI_3$. The figure shows the RDFs of the Pb-Pb, Pb-Cs, Pb-I, Cs-Cs, Cs-I and I-I atom pairs. The RDFs in the top half correspond to the Pb-rich surface (red) and the figures at the bottom half to the Pb-poor surface (blue).

7 Defective surfaces

Defective perovskite surfaces are created with vacancies. The defects in these structural models are created by removing the atoms of certain species, thus resulting in vacancies in the lattice. For the isolated defects, we create slabs with a two vacancies in total, one of each kind. As shown in Figure S7a, the top surface has a V_{Cs} defect and the bottom surface a V_I defect. For closely spaced pairs of V_{Cs} and V_I defects, Figure S7b, we create the pair of defects at the top and bottom of the slab. The slabs in the simulations are at least 50 Å thick, with the lateral dimensions at least spanning 50 Å wide.

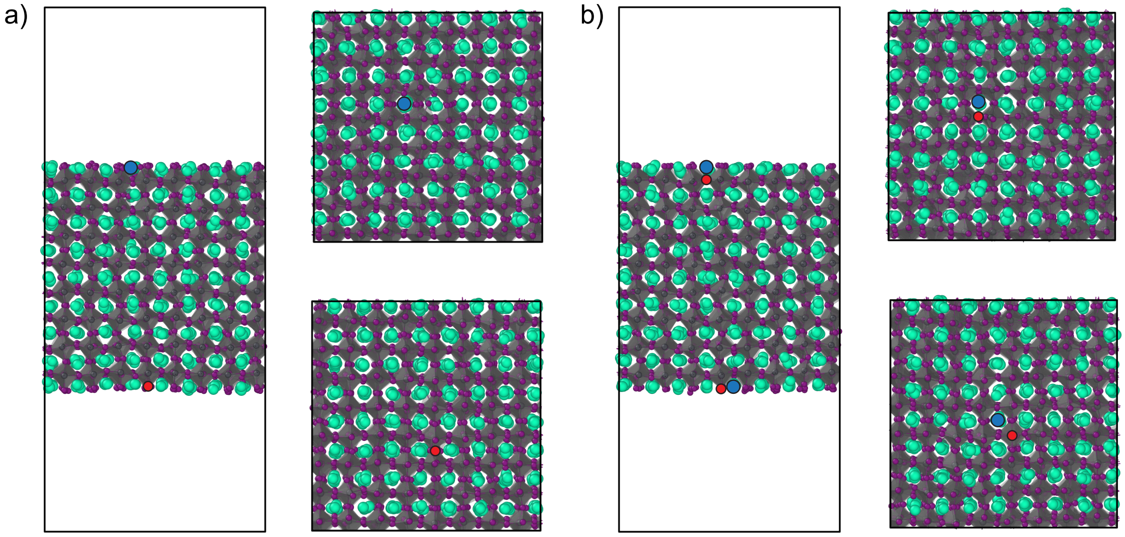

Figure S7: Structural models used to study the effect of point defects on the stability of the Pb-poor (110) orthorhombic perovskite surface. The blue and red circles are used to indicate the position of the Cs and I vacancy, respectively. (a) A slab with a single V_{Cs} at the top surface and a single V_I at the bottom surface. (b) A slab with closely-spaced defect pairs of V_{Cs} and V_I at both the top and bottom surface.

8 Grain boundary evolution

The degradation of the $5\Sigma(210)(0.4, 0)$ grain boundary using structural snapshots in the time interval ranging from 0 ps to 600 ps is explored in Figure 7. For a complete overview of all studied grain boundaries, the degradation of the $3\Sigma(112)(0.4, 0)$ grain boundary (Figure S8) and dynamical evolution of the $3\Sigma(111)(0, 0)$ grain boundary (Figure S9) are shown here.

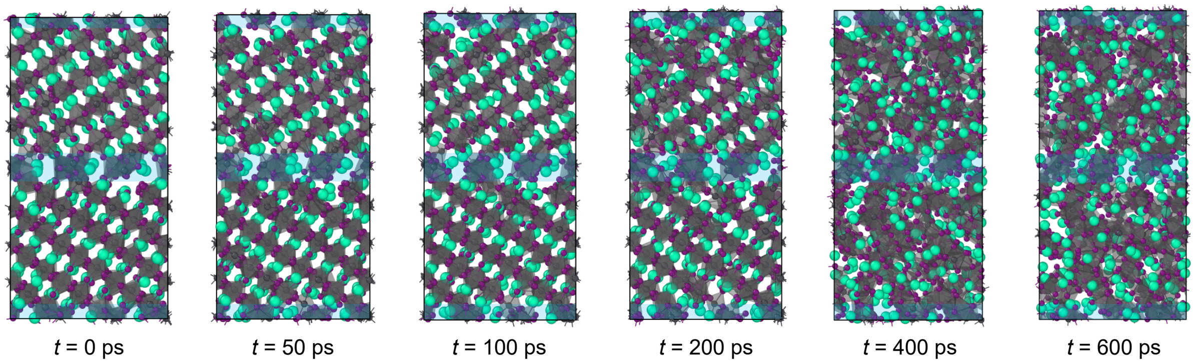

Figure S8: Degradation of a $3\Sigma(112)(0.4, 0)$ grain boundary. The blue areas highlight the grain boundaries in the structures.

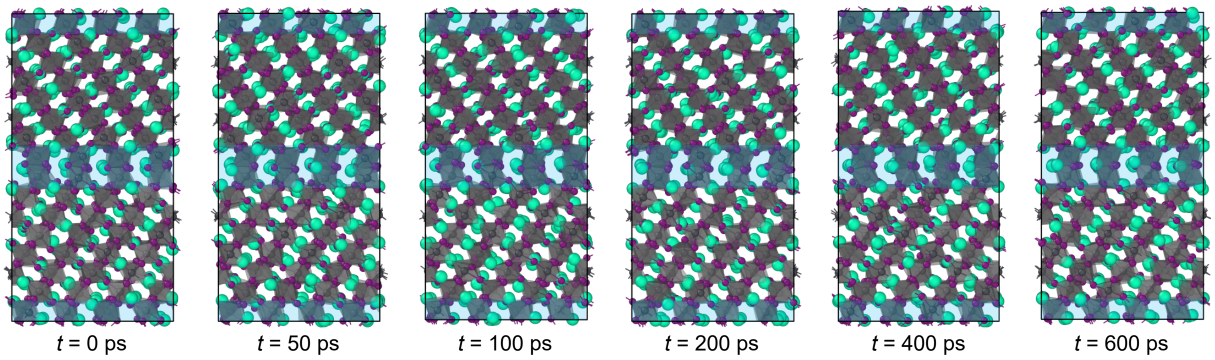

Figure S9: Dynamical evolution of a $3\Sigma(111)(0, 0)$ grain boundary. The blue areas highlight the grain boundaries in the structures.

References

(1) AMS 2021, SCM, Theoretical Chemistry, Vrije Universiteit, Amsterdam, The Netherlands, http://www.scm.com/.

(2) Guo, Y.; Wang, Q.; Saidi, W. A. Structural Stabilities and Electronic Properties of High-Angle Grain Boundaries in Perovskite Cesium Lead Halides. *J. Phys. Chem. C* **2017**, *121*, 1715–1722.

(3) Cheng, J.; Luo, J.; Yang, K. Aimsgb: An Algorithm and Open-Source Python Library to Generate Periodic Grain Boundary Structures. *Comput. Mater. Sci.* **2018**, *155*, 92–103.

(4) Seidu, A.; Dvorak, M.; Rinke, P.; Li, J. Atomic and Electronic Structure of Cesium Lead Triiodide Surfaces. *J. Chem. Phys.* **2021**, *154*, 074712.

(5) Long, L.; Cao, D.; Fei, J.; Wang, J.; Zhou, Y.; Jiang, Z.; Jiao, Z.; Shu, H. Effect of Surface Intrinsic Defects on the Structural Stability and Electronic Properties of the All-Inorganic Halide Perovskite $CsPbI_3$ (001) Film. *Chem. Phys. Lett.* **2019**, *734*, 136719.

(6) Michaud-Agrawal, N.; Denning, E. J.; Woolf, T. B.; Beckstein, O. MDAnalysis: A Toolkit for the Analysis of Molecular Dynamics Simulations. *J. Comput. Chem.* **2011**, *32*, 2319–2327.

(7) Gowers, R. J.; Linke, M.; Barnoud, J.; Reddy, T. J. E.; Melo, M. N.; Seyler, S. L.; Domański, J.; Dotson, D. L.; Buchoux, S.; Kenney, I. M.; Beckstein, O. MDAnalysis: A Python Package for the Rapid Analysis of Molecular Dynamics Simulations. *Proceedings of the 15th Python in Science Conference* **2016**, 98–105.
